# Real-time sharing of gaze data between multiple eye trackers–evaluation, tools, and advice

**DOI:** 10.3758/s13428-016-0806-1

**Published:** 2016-10-14

**Authors:** Marcus Nyström, Diederick C. Niehorster, Tim Cornelissen, Henrik Garde

**Affiliations:** 1 Humanities Lab, Lund University, Helgonabacken 12, Lund, SE 22362 Sweden; 2 Scene Grammar Lab, Goethe University Frankfurt, Theodor-W.-Adorno-Platz 6, Frankfurt am Main, DE 60323 Germany

**Keywords:** Eye tracking, Digital classroom, Shared gaze

## Abstract

Technological advancements in combination with significant reductions in price have made it practically feasible to run experiments with multiple eye trackers. This enables new types of experiments with simultaneous recordings of eye movement data from several participants, which is of interest for researchers in, e.g., social and educational psychology. The Lund University Humanities Laboratory recently acquired 25 remote eye trackers, which are connected over a local wireless network. As a first step toward running experiments with this setup, demanding situations with real time sharing of gaze data were investigated in terms of network performance as well as clock and screen synchronization. Results show that data can be shared with a sufficiently low packet loss (0.1 %) and latency (*M* = 3 ms, *M* *A* *D* = 2 ms) across 8 eye trackers at a rate of 60 Hz. For a similar performance using 24 computers, the send rate needs to be reduced to 20 Hz. To help researchers conduct similar measurements on their own multi-eye-tracker setup, open source software written in Python and PsychoPy are provided. Part of the software contains a minimal working example to help researchers kick-start experiments with two or more eye trackers.

## Introduction

Eye trackers have in the past been expensive to buy and quite difficult to operate. This situation has changed dramatically over the past decade, and an eye tracker with a full software development kit can now be acquired for less than $100^1^. Anyone with a little bit of programming skills can therefore quite easily set up and run an eye-tracking experiment. The reduction in price combined with general improvements in computer technology have made it practically feasible to run experiments with two or more eye trackers.

Even though the majority of studies where eye movements are recorded still take place in quiet rooms where participants are recorded individually, experiments with multiple participants, shared gaze, and/or gaze guidance are becoming increasingly more common, for instance to answer questions about collaboration, learning, or problem solving. For example, Nüssli (2011) found that two people who play a collaborative Tetris-game perform better when they ‘share gaze’ , i.e., when they look at the same position at the same time, or with a constant time lag. In another study investigating collaboration, shared gaze was compared with other means of communication during a visual search task (Brennan et al. 2008). Compared to no communication, communication by voice, and communication by voice and gaze, seeing where the other person looked during the search task was reported to be the most efficient means of communication in terms of search speed, but also scored well in terms of accuracy. Shared gaze seemed to encourage the participants to divide the search area between them, such that each participant had to search a smaller region. Seeing where another person looks has also been used to guide learners’ attention, and there is evidence that showing an expert’s eye movements to novices can improve learning (Jarodzka et al. 2010; Mason et al. 2015; Leff et al. 2015). In addition, seeing another person’s eye movements can “promote attentional shifts that trigger insight problem solving” (Litchfield and Ball 2011).

A multi-eye-tracker setup clearly offers exciting possibilities for future research, either as a direct extension of the research previously described, or in novel paradigms within social psychology (e.g., Strukelj et al. 2016). Several new experimental options become available with a multi-eye-tracker setup. First, and perhaps the most obvious, is to increase the throughput of participants by recording several participants at the time in each session. No additional software would be required since each system would be considered a single, isolated unit. One could also imagine a situation where it is important to have a common onset or offset of a trial, such that all participants view a stimulus or perform a task at the same time. Such an experiment would require a start command to be sent from one of the computers on the network, or to be triggered by a scheduled event based on the local system clock. Part of such an experiment could include feedback, such that information about viewers’ eye movements is summarized and presented to all participants after each trial or other experimental unit. The most demanding scenario from a system, network, and implementation point of view would be experiments with instantaneous feedback, e.g., where data are recorded and shared in real-time across multiple systems/participants.

While using a multi-eye-tracker setup is necessary to address certain research questions, there are also many issues to consider when recording more than one participant at a same time. For instance, how do I quickly distribute an experiment to all the different systems? How do I keep all the systems up-to-date, and make sure that they are identical in terms of software and hardware? Do all systems have similar performance in terms of eye-tracking data quality? How do I synchronize the recordings temporally? Besides strictly technical issues, how do you calibrate multiple participants in the same room at the same time? Do they not disturb each other? Finally, some theoretical concerns arise. Are there social effects associated with having multiple people perform a task in the same room? Even though all of these issues are important, we will focus on technical issues related to the most demanding scenarios, which require real-time sharing of gaze.

The aims of this paper are threefold. First, we want to test whether the performance of our particular setup—dubbed the *Digital Classroom*—meets the requirements for running experiments in which multiple users share their gaze data in real-time. In particular, we need to know how the eye-tracker data are affected by adding more participants or increasing the data rate. Second, we want to provide a collection of open source tools that allow researchers to diagnose their own setup as well as quickly implement experiments with multiple eye trackers and/or where participants’ eye-tracker data are shared in real time. Finally, we want to provide practical advice for people who are planning to run this type of experiments.

After describing the setup of the Digital Classroom (“The digital classroom - hardware and software”), we identify and evaluate the key components required for successful real-time sharing, display, and synchronization of gaze data: a computer’s clock resolution and drift (“Synchronizing clocks”), network performance (“Measuring network performance”), and synchronization across different screens (“Investigating the synchronization across screens”). Finally, a general, minimal working example is provided where a server script is used to control an experiment where gaze is shared and displayed in real-time across several computers during a visual search task (“Shared gaze: a minimal working example”).

## The digital classroom - hardware and software

**Fig. 1:**
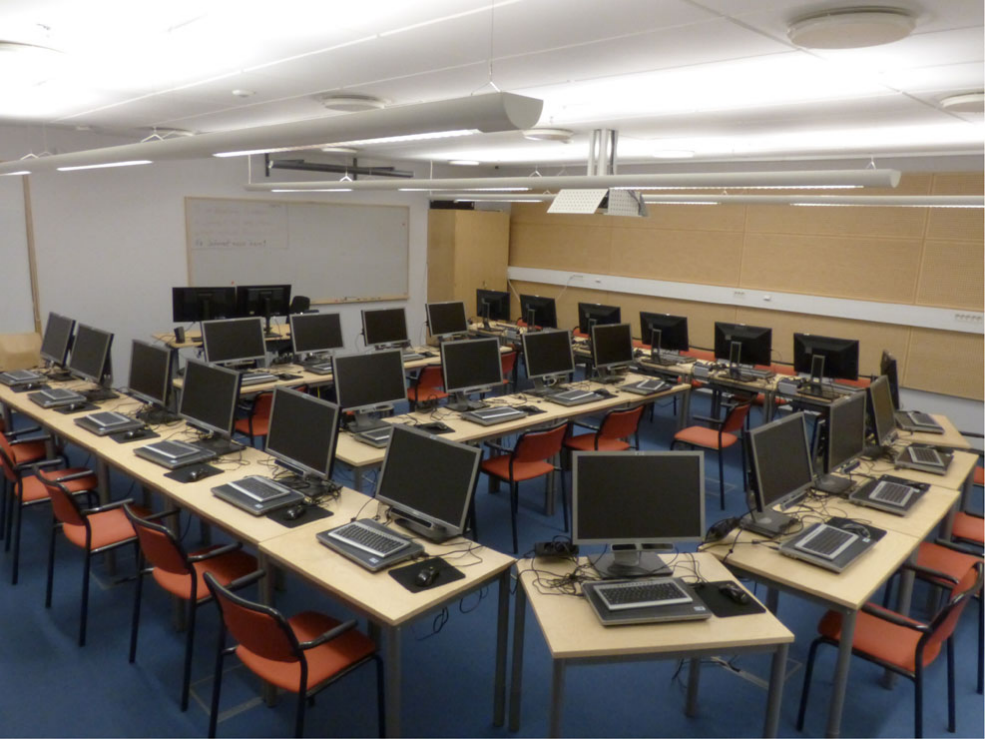
The Digital Classroom, Lund University, equipped with 25 RED-m eye trackers from SensoMotoric Instruments

The Lund University Humanities Laboratory recently acquired 25 RED-m eye trackers from SensoMotoric Instruments (SMI). The room that hosts these systems has been dubbed the Digital Classroom, and a picture of the setup can be seen in Fig. 1. Each of the 25 RED-m systems, denoted a *client*, includes a Dell laptop, a 22 in. screen, and a standard keyboard and mouse. The Digital Classroom also includes a *server* computer, which is used as a time server as well as a master to control and manage the clients. The server and clients are connected over a network through a wireless router (NETGEAR N600 Wireless Dualband Gigabit router). The full hardware and software configurations are provided in Table 1.

**Table 1:** Hardware and software configuration of the server and clients

	Server	Client
Type	Desktop	Dell Laptop
OS	64-bit Windows 7 Prof. SP 1.	32-bit Windows 7 Prof. SP 1.
Processor	Intel Core i7-4770 CPU @3.40GHz	Intel Core i7-2640M CPU @2.80GHz
RAM	16 GB	4 GB
Clock res.	1 ms (default)	15.6 ms (default). Can be set to 1 ms.
Graphics card	–	NVIDIA NVS 4200M
Network card	–	DW 1501 Wireless, N WLAN Half MiniCard, 802.11h+d
Screen	–	Dell P2210 (1680×1050 pixels, 60 Hz, 32-bit color)
Eye tracker	N/A	SMI RED-m running iView X (v. 3.2.20)
Exp. software	PsychoPy (v. 1.80.03)	PsychoPy^a^(v. 1.81.02), TimerTool^b^, SP TimeSync^c^ (v. 2.4)

^a^ www.psychopy.org

^b^ http://vvvv.org/contribution/windows-system-timer-tool

^c^ http://www.spdialer.com/timesync/

The basic infrastructure for file sharing and communication across computers is set up as follows. First, each computer is assigned a unique IP-address, where the last two digits represent the name of the computer on the network. The clients have IP addresses ranging from 192.168.1.1 to 192.168.1.25. The IP-address of the server is 192.168.1.28. Files are distributed from the server to the clients through shared network folders that are synchronized with the free and open source program FreeFileSync.^2^ Second, messages can be broadcasted from the server to the clients. The messages are received by a lightweight script running on the client side that reads and executes commands received from the network in a Windows command-line interface.

While we were restricted to the current hardware in the Digital Classroom, we could choose experimental software freely. The choice fell on Python and PsychoPy, a free toolbox to build experiments in neuroscience, psychology, and psychophysics (Peirce 2007, 2008). The main reasons are that Python is free and that there is a trend that increasingly more eye movement researchers use Python to build their experiments and to analyze their data. Open source, python-based software specific to eye movement research is beginning to emerge on several fronts, e.g., PyGaze (Dalmaijer et al. 2014), and Sol Simpson’s ioHub (now integrated with PsychoPy). Through the socket-library, Python also offers an easy and intuitive way to manage the network communication across different computers. Critically, the socket-library supports multicasting, which can be used to limit bandwidth consumption by including only a desired subset of the clients in an experiment.

Software to measure network latencies as well as running the visual search demo are provided on GitHub (https://github.com/marcus-nystrom/share-gaze.git).

## Synchronizing clocks

There are several good reasons to keep the clocks on the different computers synchronized. First, it brings order to the data-files during later analysis, so the researcher can track when the data were collected and in what order, and use this information when sorting and pre-processing the data. Second, when it is important that a trial starts in synchrony for several participants, it is usually more accurate to use the client’s local synchronized clock, then to rely on a start command sent over a network with variable delays. Finally, synchronized and drift-free clocks make it easier to evaluate network delays, since the timestamps from different computers can be compared and subtracted directly.

While the benefits of synchronizing the clocks on the clients in the classroom seem clear, they are of practical use only if the synchronization can be performed with sufficient accuracy. Perhaps the most common way to synchronize computer clocks is against a time server using the Network Time Protocol (NTP).^3^ The protocol is designed to synchronize clocks across variable-delay networks and can reach accuracies below one milliseconds between peers over a local network. Besides accuracy, the NTP protocol reports roundtrip delay between the server and the client.

A prerequisite for being able to measure network transfer delays and to synchronize the clocks with high accuracies is a high resolution clock. Clock resolution is defined as the interval with which the system clock is updated, and thus corresponds to the smallest time difference that can be measured by the system. For most modern PCs, the default resolution is 15.6 ms, i.e., 64 Hz.^4^ From the point of measuring network latencies, this means that latencies smaller than 15.6 ms will be reported as 0 ms and therefore cannot be measured accurately.

Fortunately, the clock resolution can easily be checked and modified using a number of open source libraries and tools such as the TimerTool. With TimerTool, we can set the clock resolution of the computers in our setup to anywhere between 1 ms to 15.6 ms through a simple command line argument. For newer computers, a resolution of 0.5 ms may be possible. For instance, the command


C:\PathToTool\TimerTool.exe-t1-minimized


sets the resolution to 1 ms and starts the tool minimized to system tray. The disadvantage of increasing the granularity of the clock is that it increases the CPU load of the computer as background processes are activated more frequently by the computer’s operating system.

### Methods

To synchronize the clocks on the clients in the Digital Classroom, we first set up the server computer as a time server (cf. GitHub repository for details). A Python script


server_sync_clock.py


was executed on the server to start another Python-script


client_sync_clock.py


locally on each client to perform the actual synchronization by calling the freely available software SP Timesync. The offset in the clocks directly after synchronization was measured 100 times with the Python NTP library (ntplib v. 0.3.3). In parallel to SP Timesync, we also tried to set the clocks on the clients with a win32api call from Python (SetSystemTime), but abandoned this method due to its significantly poorer accuracy as this method did not correct for network latencies like the NTP protocol does.

To prevent unwanted influence of network load on the synchronization accuracy, the computers communicated with the time server one at the time. Before the clocks where synchronized, the clock resolution was changed from 15.6 ms to 1 ms using TimerTool. A check was also performed to measure the actual resolutions before and after the change using two different clocks available in Python through the time module. The check consisted of a for-loop that requested a timestamp every millisecond over 100 iterations. Finally, we measured the clock drift on the clients by checking the offset against the time server once every hour during 24 hours.

### Results & discussion

Four main questions were of interest. First, did the call to change the clock resolution work, i.e., did it provide an average measured resolution of one millisecond with a low standard deviation? Second, how accurately can we synchronize the clocks against the time server? Third, how large is the drift, informing us about how often we need to synchronize the clock? Finally, can all clients reach the same resolution and accuracy, or is there individual variation?

**Fig. 2:**
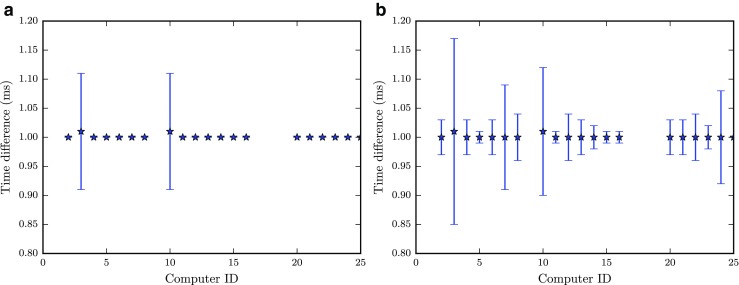
A check that one millisecond time differences can be measured after setting the clock resolution to 1 ms. Two ways to get timestamps were tested: **a** time.time and **b** time.clock. Error bars represent standard deviations around the mean for 100 measured differences

Figure 2 shows the average difference between timestamps requested every millisecond by (a) time.time and (b) time.clock. Both methods provide average differences close to the desired value of one, and the standard deviations are at most 0.2 ms. The difference between using time.time and time.clock seems negligible, even though the former provides slightly lower standard deviations. This is most likely due to the lower granularity of time.time on Windows.^5^

**Fig. 3:**
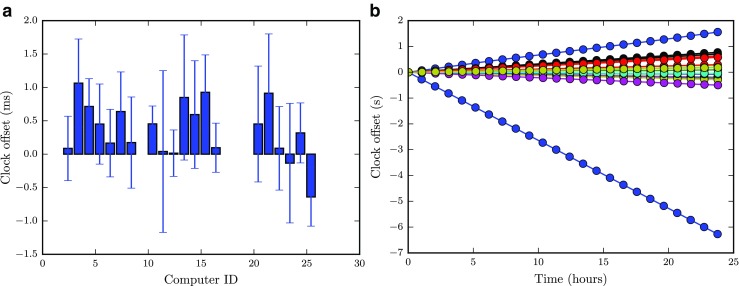
Results of checking the clock offsets (*M*±*S* *D*) 100 times directly after a synchronization (**a**) and once every hour over a 24-hour period (**b**)

Clock accuracy measured directly after synchronization can be seen in Fig. 3a. The mean offsets are within one millisecond, and the precision of the measurements is high, typically also within a millisecond. There is some individual variation, but too small to have any practical relevance for our purposes. Changing the clock resolution from 15.6 ms to 1 ms decreases the average of absolute values across all clients from 3.19 ms (*S* *D* = 2.60) to 0.44 ms (*S* *D* = 0.33). This increase highlights the importance of having a sufficiently high clock resolution to be able to reliably measure and correct clock offsets.

Running additional checks of the clock accuracy during a 24 hour interval reveals that there is significant clock drift, in particular for some of the clients (Fig. 3b); in the worst case scenario, the drift can be up to several seconds over this relatively short period of time. Since the drift is largely linear, the worst case corresponds to a rate of 262 ms/hour. Although the drift is measured against an arbitrary reference—the clock on the server computer—the test allows us to determine how the clients drift relative to each other. The maximum offset between two clients reaches more than 7.5 seconds over the 24 hour period.

In summary, the clocks on the clients can be synchronized to the server computer with a near millisecond accuracy and precision, given that the clock resolution is increased to 1 ms compared to the default 15.6 ms. The variation across computers is small directly after the clocks have been set, but the drift rates seem largely system dependent. If synchronized clocks are important for your setup, the clocks should be synchronized at least at the beginning of every experiment run, but preferably at the onset of each trial.

## Measuring network performance

The next concern is to quantify how much time it takes to send gaze data from one client to the other, i.e., the latency, and also whether these data arrive at a constant rate, i.e., the variance in latency. The latter will be denoted reliability. Knowledge of the latency helps decide what kinds of experiments are possible with your setup. For some tasks, such as collaborative tasks requiring tight synchronization of the participants’ actions, anything but minimal latency may be unacceptable. Yet for other tasks, latencies in the hundreds of milliseconds may be sufficient to carry out your experiment. Similarly, requirements on packet loss and reliability can vary markedly. While for some experiments it would not matter if a sizable amount of gaze data never arrive at the other client, and that they arrive at variable intervals, other experiments may require all data to arrive and to do so at a regular rate. As such, an important step in planning your experiment is to characterize the latency, reliability, and packet loss when sending gaze data between clients in your setup.

While sharing gaze data between two clients may be easy, having multiple clients each send their data to all the other clients might put a significant load on the network. In this section we evaluate the performance of our setup in terms of average end-to-end latency, variance in latency (reliability), and the proportion of lost packets. We examine how these measures are affected by the number of clients that are communicating with each other and the rate at which they send data.

### Methods

#### Experimental setup

In this experiment, each active client sends its gaze data to all other active clients. Between two and 24 clients were active in each run, and sent their data at a common rate of 60 Hz, 30 Hz, 20 Hz, 15 Hz, 10 Hz, 6 Hz, 4.3 Hz, or 3.3 Hz. Four runs with two and three clients were performed at each data rate. Three runs were conducted when four to six clients were active, while two runs were performed when seven or more clients were active. More runs were collected when only a few clients were active to ensure sufficient data points.

Each run was started by a script running on the server computer


server_CastThread.py


that started a script on the active clients with a call, e.g.,


client_CastThread.py sim 10 1 0 1 1 1000


The parameters, in sequential order, control

- whether simulated (sim) or eye-tracking data (iView) are used
- the rate at which data are transmitted in fractions of the screen refresh rate (10 means every 10*th* screen refresh, i.e., 60/10=6 Hz)
- whether or not to receive one’s own sent data
- whether the clients should start directly or wait for a start command from the server
- whether the send rate should be locked to the screen flip or to the system clock
- whether or not received data should be visualized on the screen
- the number of packages to be sent, which determines the duration of a run together with the data rate.

During each run, between 1000 and 3000 packets were sent by each client through a Python socket. Packets were sent over UDP using multicast, such that each packet only had to be sent once, and the router forwarded it to the other active clients. Each packet sent from client *i* consisted of the tuple (*k*_*i*_,*x*_*i*_,*y*_*i*_,*t*_*i*, *S*_), where *k* represents the sequence number, (*x*_*i*_,*y*_*i*_) simulated gaze coordinates, and *t*_*i*, *S*_ a timestamp indicating the local machine time at which the packet was dispatched. To ensure optimal throughput, data were received in a separate thread using a blocking Python socket. When a package arrived at client *j*, a local timestamp of its arrival, *t*_*j*, *R*_ was added to the tuple and was stored in a text file along with the IP address of the client from which the packet was sent. Since the scripts did not start at exactly the same time after the call from the server, data from the clients were not transmitted in bursts over the network.

It should be noted that while we used simulated gaze data in the measurements reported here, the results were identical when we sent data acquired in real time from the RED-m eye tracker connected to each client; that the extra load coming from the eye tracker did not affect the performance is probably due to the modern multi-core processor technology present in our laptop machines, which allows efficient multitasking.

**Fig. 4:**
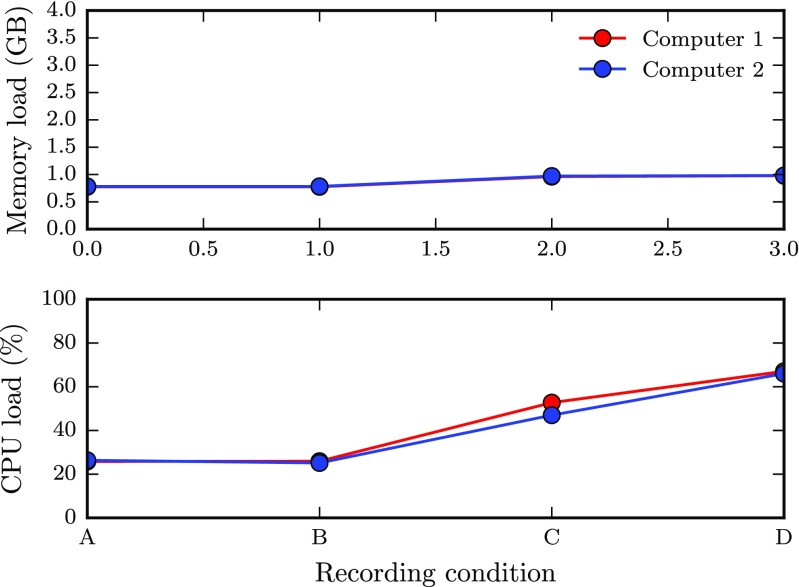
Influence of clock resolution and eye tracking on memory-, and CPU load when running the find Wally demo for 60 s. *A*: 15.6 ms clock resolution, eye tracker not running; *B*: 1 ms clock resolution, eye tracker not running; *C*: 1 ms clock resolution, eye tracker running; *D*: 1 ms clock resolution, participant being eye tracked. The data were recorded from two computers

Figure 4 exemplifies how memory and CPU-load are influenced when changing the clock resolution from the default 15.6 ms (A) to 1 ms (B). The last two recording conditions correspond to when the eye tracker is running but no participant is being tracked (C), and when an actual participant is being tracked (D). As can be seen from the figure, changing the clock resolution has a very small influence on the load. Running the eye tracker increases both memory and CPU-load, which increase even further when a person is being tracked. Importantly, even in the most demanding situation, neither the memory nor the CPU-load are close the their maximum values, which correspond to the largest values on the y-axes. The data were acquired with the standard Performance Monitor Windows-tool while running the provided demo-script (find Wally) on two different computers.

#### Data analysis

The first measure of interest is the end-to-end latency *λ*_*ij*_ of our setup, i.e., the amount of time that elapsed between when a packet was sent from client *i* until it was received on client *j*. A simple strategy to compute the latency would be to subtract the sent timestamp *t*_*i*, *S*_ from the received timestamp *t*_*j*, *R*_. However, the result would be valid only if the clocks on the two clients were synchronized exactly and did not drift. As we saw from the measurements in “Synchronizing clocks”, this is not the case. Hence, a more flexible analysis strategy was devised, which we will illustrate by means of a thought experiment.

Consider the hypothetical case where the latency is zero and the relative clock offset *ℓ* between clients *i* and *j* is 500 ms, with client *i* having an earlier clock time than client *j*. In this case, timestamps *t*_*i*, *R*_ received at client *i* would lag 500 ms behind the timestamps *t*_*j*, *S*_ sent from client *j*, i.e., Δ*t*_*ji*_ = *t*_*i*, *R*_−*t*_*j*, *S*_ = −500 ms. Conversely, packages sent in the other direction would have the same time difference but with an opposite sign, Δ*t*_*ij*_ = *t*_*j*, *R*_−*t*_*i*, *S*_ = 500 ms. Adding a processing and network delay of 20 ms increases both differences: Δ*t*_*ji*_ now becomes −500+20=−480 ms and similarly Δ*t*_*ij*_ = 500+20=520 ms.

As can be seen from the results of this thought experiment, processing and network delays between two clients causes a shift in the *average* difference (Δ*t*_*ij*_+Δ*t*_*ji*_)/2, and the size of this shift equals the latency *λ* we want to measure. If $\lambda _{i,j}^{k}$ denotes the average latency of the *k*th packet, the average latency for a whole run, $\bar {\lambda }_{i,j}$, can be computed as

1 $$ \bar{\lambda}_{i,j} = \frac{1}{N}\sum\limits_{k=1}^{N}\lambda_{i,j}^{k} $$

where *N* is the number of packets that was sent by each client. It should be noted that this measure of latency is not sensitive to clock drift, as drift only causes a symmetric expansion of time differences, Δ*t*, around $\bar {\lambda }_{i,j}$.

**Fig. 5:**
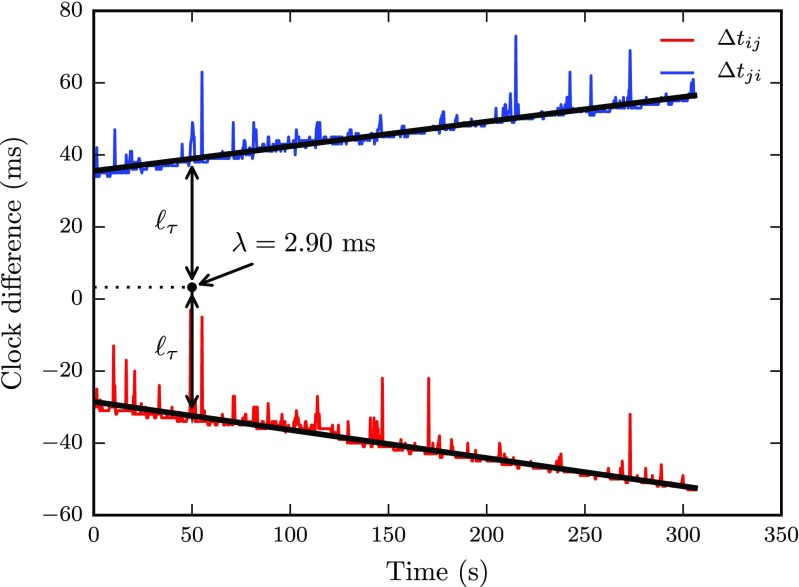
Time from a packet is sent from client *i* until it arrived to client *j*, Δ*t*_*ij*_ (*red*) and vice versa, Δ*t*_*ji*_ (*blue*). *Black lines* represent robust linear fits of the data. All timestamps represent differences between local clock times. The *lines* are symmetric around the estimated latency, *λ*. The differences are not constant over time since the clocks drift relative to each other. The clock offset at time *τ* is represented by *ℓ*_*τ*_. The data were recorded in a run with five clients sending data at a rate of 3.33 Hz

**Fig. 6:**
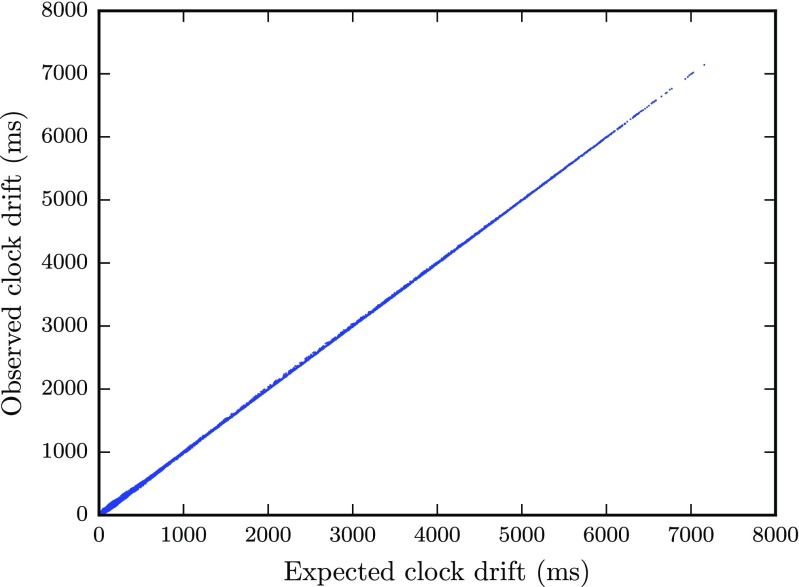
Clock drift measured with the Python NTP-library in Fig. 3 **b** plotted against the clock drift observed with the method described in this section

We used a slightly more complicated method to compute latencies as we ran into additional problems with our timestamp data. First, there are outliers among the Δ*t* time series which cause the latency to be overestimated. Second, due to differences in the clock drift rate, some of the clients finished slightly earlier than others and did not record some of the last samples sent by the slower clients, which would also lead to a bias using the above method. Third, packet loss occurs in almost all trials and would also lead to bias using the above method. Therefore, for each pair of clients we fit a line to the two Δ*t* time series using a robust regression procedure with Matlab’s robustfit function, using the default Huber distance norm. Following the logic of the method above, end-to-end latency was given by the mean of the intercepts of the lines fit to the data for each pair of clients. An example of this procedure is given in Fig. 5, which present data from a run with five clients sending packets at a rate of 3.33 Hz. Besides latency computed from the intercepts, the slopes of the lines reflect the clock drift. Moreover, the relative clock offset at time *τ* equals in this example *ℓ*_*τ*_ = 32.4 ms. As a measure of reliability, the variance of the residuals of the line fit was calculated by means of the mean absolute deviation (MAD), which is a robust estimator of the standard deviation. Since the packets sent by each client had sequence numbers, packet loss was determined simply by computing the proportion of missing sequence numbers. Figure 6 verifies that the clock offset determined with this method closely matches that predicted by the total clock drift during 24 hours presented in Fig. 3b, using the Python NTP-library.

### Results & discussion

**Fig. 7:**
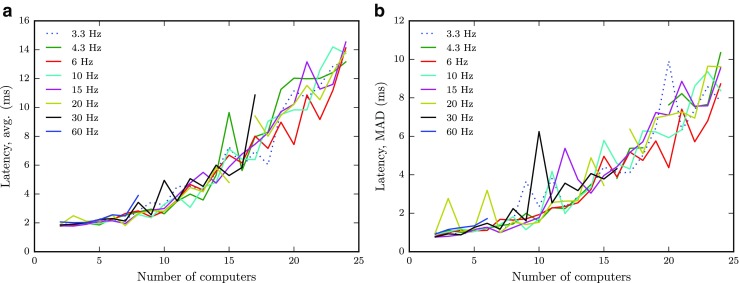
Mean (**a**) and MAD (**b**) of latency as a function of the number of clients for each data rate (3.3 → 60 Hz, see legend)

Figure 7a shows the average latency between clients as a function of the number of clients that was active simultaneously, and the tested packet rates. When the network is no longer able to handle the number of packets sent by the clients, the distribution of latencies between pairs take on a bimodal shape as some clients receive data at lower rates than others. This happened when 9 or more clients transmitted data at 60 Hz, and for 18 computers or more at the 30 Hz data rate. As mean latencies become unreliable in this case, we do not plot data for these cases in Fig. 7a. The figure shows that latency increases at a slightly increasing rate with the number of clients, from just below 2 ms to about 16 ms. Interestingly, the packet rate did not affect the latency until it reached the limit where the network could no longer handle the load.

As a measure of reliability, Fig. 7b shows the variability in latency. The trend is similar to the latency, with values of about 2 ms when only a few clients are active, and values reaching up to 9 ms when all available clients are used. Again, the different data rates did not affect the variability.

**Fig. 8:**
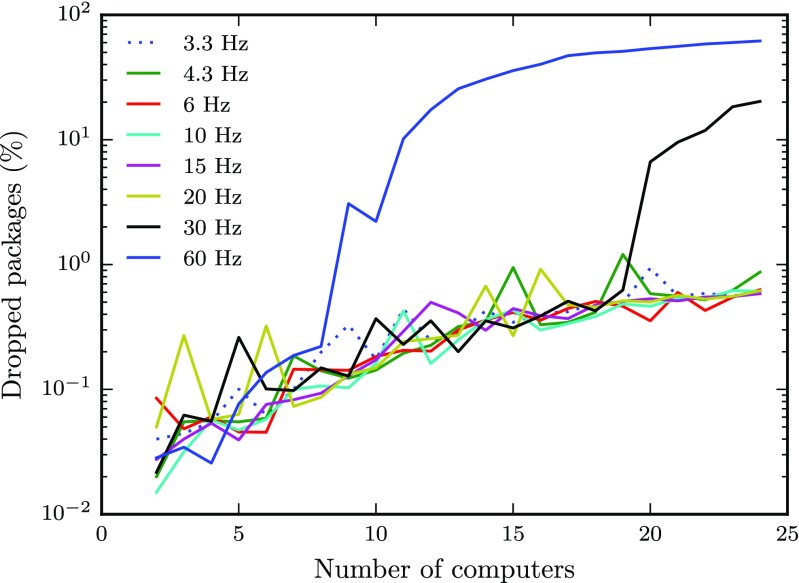
Percent dropped packages as a function of the number of clients for each data rate. Note the log-scale on the *y*-axis

Figure 8 shows the proportion of packet loss on a logarithmic scale. The packet loss is less than 0.1 % when only a few clients are active and levels out at around 1 % as more clients are included. However, the data also show that above a certain packet rate, the proportion of dropped packets increases rapidly under certain conditions. For instance, when nine clients were sending data at 60 Hz to each other, the proportion of dropped package increased rapidly, and continued up to almost 60 % as more clients were included. Similarly, when about twice as many clients (20) were active at half the packet rate (30 Hz), the proportion of dropped packages showed a similar behavior. This sharp increase in packet rate is a further indication that the network could no longer handle the number of packets sent by the clients.

It should be noted that while the amount of packets received by each client depends linearly on both the number of clients transmitting and the rate at which the packets are sent, the amount of packets that travel across the network depends linearly on the rate at which clients send the data, but increases quadratically as more clients are added to a run. As such, if it was the rate at which data could be received by a client that limited the latency and transmission reliability observed at each client, we would expect the latency and variance to increase linearly as more clients were added to a run. Instead, observed latencies and variance increased quadratically, suggesting that the limiting factor in our setup is the amount of traffic at the router. As such, using professional level cabled network equipment may remove the limits and mitigate the increases in latency we observed.

Our tests reveal that even with a Python socket running over a consumer grade WiFi network, performance is sufficient for almost all configurations we tested. However, in the most demanding cases with many computers and high packet rates, the setup with the WiFi network fails.

## Investigating the synchronization across screens

Beside the sampling rate of the eye tracker and the capacity of the network, the time it takes to show one participant’s gaze data on another participant’s screen depends on the refresh rate of the monitor. Since we are using 60 Hz screens, it takes in the worse case scenario more than 16.7 ms from the time the gaze data are received until ithey can be presented on the screen. This is because the gaze data may arrive after the command to render the next screen has been issued. Techniques that enable issuing the rendering command as late as possible and thereby ensuring that the newest data are displayed have previously been developed for gaze-contingent displays (e.g., Aguilar and Castet^2011^). However, this can reduce but not eliminate this problem. In a situation with shared gaze, where data from one client are to be displayed on each of several other clients’ screens, any asynchrony between the different screen refresh cycles will inevitably cause the data to be displayed at different times, even in the case where the data arrived at the same time.

### Methods, results & discussion

To test whether the screen refresh cycles of multiple screens were synchronized we put three screens next to each other, and changed the screen color from black to white every refresh. The screens were filmed with a CASIO EX-ZR800 digital camera at 480 Hz and a resolution of 224 ×160 pixels.

**Fig. 9:**
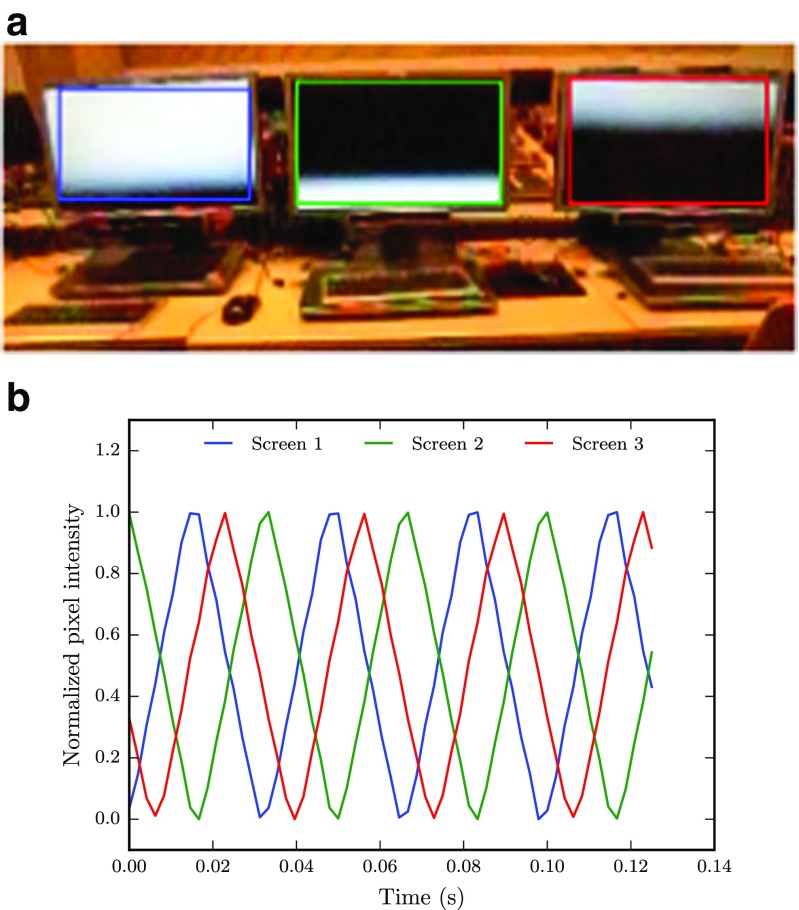
**a** Cropped frame from the video of three screens changing from *black* to *white* every screen refresh. The screens operate at 60 Hz and the video is recorded at 480 Hz. **b** Average, normalized pixel intensities extracted from areas enclosing each of the three screen region in the video frame in (**a**). A value of 0 means that the entire area within a *box* consists of *black pixels*, whereas a values of 1 refers to a completely white screen

Figure 9a shows how the screens are updated and indicates that each screen is at a different stage in the refresh cycle. To quantify this behavior more precisely, the content of a box enclosing each screen in the video is extracted and analyzed. As seen in Fig. 9b, the average normalized pixel intensity within each box peaks at different locations, and clearly indicates the difference in phase. Consequently, regardless of the network and other processing delays, the screen refresh rate as well as the temporal synchronization between different screens are important bottlenecks when precise timing of stimuli or real-time gaze sharing is required. Since we have 60 Hz screens in our setup, a gaze sample that arrives to two clients at the same time could in the worst case be displayed on the participants’ screen with a time difference of more than one refresh cycle (16.7 ms).

## Shared gaze: a minimal working example

The purpose of this section is to provide a minimal working example that allows researchers to quickly set up their own experiments with multiple eye trackers. The demo comprises a visual search experiment and consists of a server script (wally_server.py) controlling the main flow of the experiment along with a script that runs on each client (wally_client.py). Two classes are used to handle the communication; one to communicate with the eye tracker (iview_SDK.py) and one to multicast data across the network (CastThread.py). When running the demo, a participant is instructed to find Wally—a well known character from a series of children’s books—while seeing his own and all other peoples’ gaze locations on the screen in real-time, where each participant’s gaze is indicated by a unique color. The server script launches the scripts on the clients, controls when to start the calibration and the data visualization, and makes sure that all clients calibrated successfully. The demo follows the following structure:

### Server

1. Start client scripts
2. Send command to initiate calibration
3. Wait for all clients to finish their calibrations
4. Send start command to initiate the experiment
5. Wait for all the clients to finish or until the maximum trial duration is reached.

### Client

1. Script started by call from server
2. Wait for server-command to start calibration
3. Calibrate eye tracker
4. Wait for server-command to start experiment
5. For each screen refresh
  - Read eye tracker data
  - Multicast eye tracker data
  - Read newest data from each client since last refresh (including own)
  - Draw the search image along with all received gaze data
6. Send message that the experiment has finished along with the search time to the server.

**Fig. 10:**
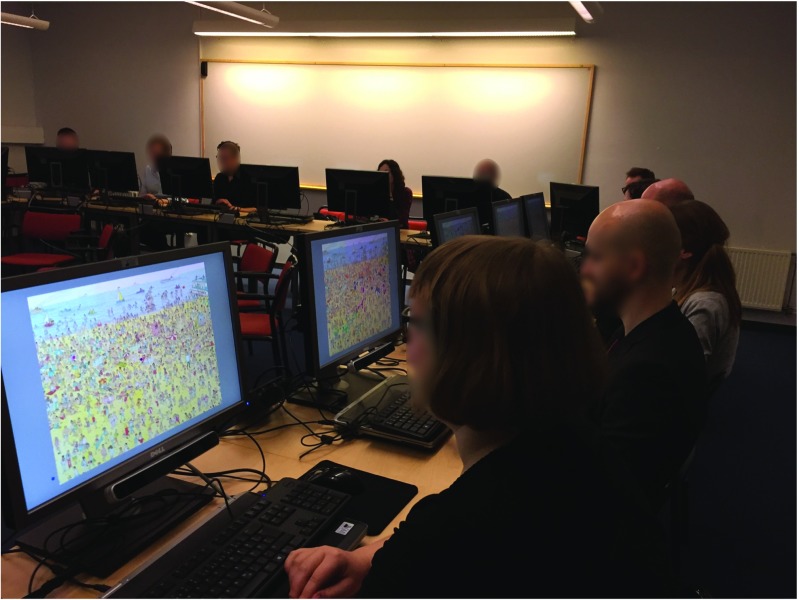
Participants searching for Wally while seeing their own and other participants’ gaze positions overlayed on the picture in real time

The classes in iview_SDK.py and CastThread.py read data from the eye tracker and the socket, respectively, in separate threads. Figure 10 illustrates when the demo is tested on 11 participants searching for Wally while seeing where the other participants look.

Even though no objective numbers are extracted from this demo experiment, one can quickly get a subjective impression of whether the setup works in the sense that no visible lag between your eye movements and the dot representing the gaze direction on the screen is discernible.

The visual search demo on the client has two parameters that control the degree of server control and whether real or simulated data are used, giving four possible combinations in total. In the simplest case, the call


wally_client.py 0 0


starts the demo on each active client, and simulated, shared data are displayed. The demo runs for of maximum time of 60 s or until it is terminated by a key press on the client’s keyboard. When both parameters are set to 1, the server and client scripts behave according to the enumerated lists above, calibrating each participant and recording and transmitting real eye movement data.

## General discussion

After acquiring and connecting multiple eye trackers over a wireless network, we wanted to know whether the setup was good enough to run experiments where gaze data are shared across participants in real time. A number of scenarios were identified (cf. the Introduction), each putting different demands on the performance of the setup in terms of the sampling rate of the eye tracker, and how quickly and reliably the data should be acquired from one computer and transmitted and displayed on other computers. The critical part of the setup required for such an investigation were identified; we adjusted, synchronized, and evaluated the clocks on the client computers, tested end-to-end latencies as a function of eye tracker sample rate and the number of eye trackers sharing gaze data, investigated the influence of screen refresh rate, and provided a demo for real-time gaze sharing.

The main result was that—even with our consumer grade wireless router—data could be shared quickly and reliably across several computers at a rate equal to the refresh rate of the screen (60 Hz). More specifically, real-time sharing of data between 8 computers was practically feasible at 60 Hz; the proportion of lost packets was about 0.1 % and the latency was lower than 3 (*M* *A* *D* = 2) ms. At 30 Hz, the number of computers could be doubled with a similar performance. However, it should be stressed that the setup with the WiFi network fails at high packet rates (30/60 Hz) when the number of computers is large (> 17, 30 Hz, > 8 60 Hz). Critically, the proportion of lost packets increases to over 50 % for the most demanding cases.

The clocks could be synchronized against the time server to within millisecond accuracy and precision. While this is likely to be sufficient for most purposes, some of the computers drift with rates up to several hundreds of milliseconds every hour. Clearly, the clocks should be synchronized at least before each experiment and perhaps before each trial just for the purpose of offline file and data sorting. When a higher degree of synchronization accuracy of data is required, one could use methods similar to those presented to calculate latency. In fact, the timestamps we collect provide a continuous measure of relative clock offsets that can be used to align eye-tracking data after the recordings are completed. It should also be mentioned that an approach similar to that proposed by Nüssli (2011) could be used for offline synchronization of eye-tracking data, where timestamps from the server are sent to all clients at regular intervals throughout the recording, and stored as messages in the data files. However, such an approach would increase the amount of traffic on the network. In addition, a timestamp sent from the server may due to the variable network delay not reach the clients at the same time. Importantly, if synchronization of client and server data is desired using this method, the network delay needs to be measured and compensated for, e.g., using a solution similar to the NTP.

Even though measures have been taken to keep all the clients as similar as possible in terms of hardware and software, it is evident their performance can differ; the clocks of some clients could always be synchronized to within milliseconds accuracy and precision, whereas others could not. Clock drift rates differed significantly, and some client remained in sync with each other while others drifted apart significantly (cf. Fig.3b). Since eye trackers are produced in much smaller volumes, have vastly fewer users than consumer style laptops, and lack a generally accepted evaluation protocol, the results across eye trackers may vary to even greater extent. It has become evident from the experiments conducted in the Digital Classroom so far that some of our systems have become preferred over others, mostly based on a subjective feeling that one works better or more reliably than the other. Quantifying such differences in terms of accuracy, precision, and latency of the eye-tracker data for each client is perhaps a natural next step.

One of the biggest challenges in this paper was to restrict the scope of the paper, and avoid the temptation to keep implementing small improvements, and try different methods and tools. There are still several factors that could be improved and tweaked in our setup with respect to the results we present. Perhaps the most obvious improvement would be to replace the wireless network with a wired network. This would most likely make it feasible to run all eye trackers at 60 Hz while retaining low latencies and proportion of lost packages. Another improvement would be to implement time critical functions, e.g., those for socket communication, in a faster programming language such as C. Buying screens with shorter response times and higher refresh rates would remove a significant source of delay in the system when display of shared gaze data is desirable. Finally, it is an open question how a solution where all communication goes via the server instead of the clients communicating directly with each other would change the results. Given the many components that could influence the performance, we would like to stress that our results are specific to our setup, and the software and hardware that were available at the time of our data collection. Therefore researchers are strongly encouraged to evaluate their own setups with the tools provided in this paper.

Although infrastructures similar to our Digital Classroom in Lund are starting to emerge, there are currently no commercial software packages that allow experiments along the lines of the demo we provide. The open source software we provide should work out of the box with eye trackers from SMI using iView X and the SMI SDK. However, given the many Python packages that allow communication with eye trackers from other manufacturers (e.g., ioHub and PyGaze, Dalmaijer et al. 2014), adapting the code to work with other known brands should be straightforward for an eye movement researcher with moderate experience with Python programming and from using a standard one-computer setup. Importantly, the demo scripts are modular and one eye tracker could easily be swapped out for another without affecting the code for the demo logic or the data transmitting and receiving across the network.

While the main focus of this paper is real-time sharing of gaze data, many of the problems we encountered during implementation and testing were related to the infrastructure of the Digital Classroom. One time consuming issue concerns the fact that the wireless network cards do not support wake on LAN, which means that we have to start each recording day by manually waking up all computers. A second practical issue is to make sure that the software and hardware are up-to-date, working properly, and are similar across all the clients. This is particularly important when the facility is an open resource for researchers across a University, as is the case at our laboratory in Lund.

**Fig. 11:**
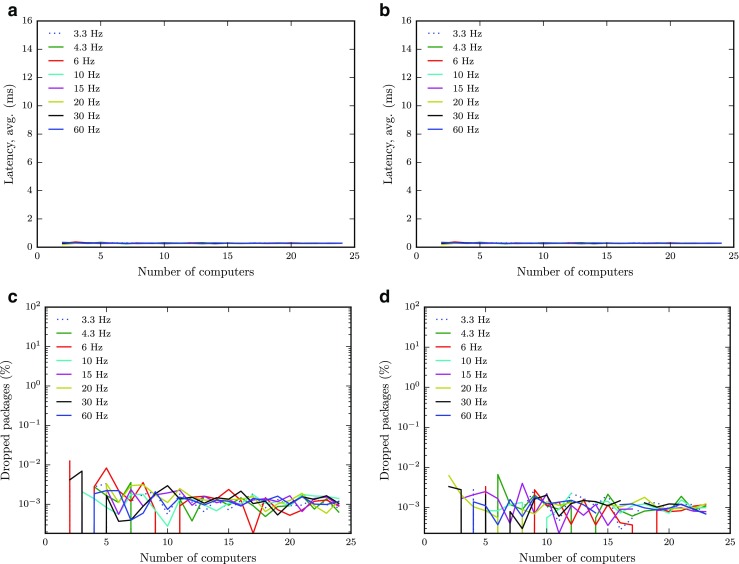
Results reproduced with the wired setup. Mean latency (*first row*) and proportion of dropped packages (*second row*) as a function of the number of clients for each data rate (3.3 → 60 Hz, see legend). Results without (*first column*) and with (*second column*) the eye tracker running are included

As a final note, we have upgraded the Digital Classroom since the first version of this paper was submitted. The setup is now wired and the switch upgraded to a Cisco SG500-52P, and in Fig. 11 results for latency (a,b) and the proportion of dropped packages (c,d) from Figs. 7a and 8 are reproduced—both with and without the eye-tracker software running in the background. Clearly, wiring the setup completely removes the problems with dropped packages and high latencies associated with the wireless setup; latencies well below 1 ms and packet losses lower than 0.01 % are reported consistently across all tested conditions. Despite the added CPU-load caused by running the eye-tracker software (cf. Fig. 4), there are only very small differences in the results.

## Conclusions

Our setup, comprising 25 RED-m systems connected through a consumer grade wireless router is, besides the most demanding cases we tested, suitable to run experiments that allow several people to share their gaze data in real-time. After wiring the setup, even the most demanding cases had very low latencies (<1 ms) and proportion of dropped packages (<0.01 %). We envision that the results, software, and advice provided in this paper will be helpful for other eye movement researchers who are thinking about setting up a Digital Classroom with multiple eye trackers, have already acquired the systems and want to investigate the performance of their setup, or are about to implement the novel experimental paradigms that this new type of setup opens up for.

## Footnotes

1
https://theeyetribe.com/

2
http://www.freefilesync.org/

3
https://tools.ietf.org/html/rfc5905

4
https://msdn.microsoft.com/en-us/library/windows/desktop/dn553408(v=vs.85).aspx

5
https://docs.python.org/2/library/timeit.html
